# Mutations in the HPV16 genome induced by APOBEC3 are associated with viral clearance

**DOI:** 10.1038/s41467-020-14730-1

**Published:** 2020-02-14

**Authors:** Bin Zhu, Yanzi Xiao, Meredith Yeager, Gary Clifford, Nicolas Wentzensen, Michael Cullen, Joseph F. Boland, Sara Bass, Mia K. Steinberg, Tina Raine-Bennett, DongHyuk Lee, Robert D. Burk, Maisa Pinheiro, Lei Song, Michael Dean, Chase W. Nelson, Laurie Burdett, Kai Yu, David Roberson, Thomas Lorey, Silvia Franceschi, Philip E. Castle, Joan Walker, Rosemary Zuna, Mark Schiffman, Lisa Mirabello

**Affiliations:** 1 Division of Cancer Epidemiology and Genetics, National Cancer Institute, National Institutes of Health, Rockville, MD USA; 2 Cancer Genomics Research Laboratory, Frederick National Laboratory for Cancer Research, Frederick, MD USA; 3 Infections and Cancer Epidemiology Group, International Agency for Research on Cancer, 150 Cours Albert Thomas, 69372 Lyon, Cedex 08 France; 4 Women’s Health Research Institute, Division of Research, Kaiser Permanente Northern California, Oakland, CA USA; 5 Departments of Pediatrics, Microbiology and Immunology, and Obstetrics & Gynecology and Women’s Health, Albert Einstein College of Medicine, Bronx, NY USA; 6 Department of Epidemiology and Population Health, Albert Einstein College of Medicine, Bronx, NY USA; 7 Sackler Institute for Comparative Genomics, American Museum of Natural History, New York, NY USA; 8 Regional Laboratory, Kaiser Permanente Northern California, Oakland, CA USA; 9 CRO Aviano National Cancer Institute IRCCS, Aviano, Italy; 10 University of Oklahoma Health Sciences Center, Oklahoma City, OK USA

**Keywords:** Cervical cancer, Human papilloma virus

## Abstract

HPV16 causes half of cervical cancers worldwide; for unknown reasons, most infections resolve within two years. Here, we analyze the viral genomes of 5,328 HPV16-positive case-control samples to investigate mutational signatures and the role of human APOBEC3-induced mutations in viral clearance and cervical carcinogenesis. We identify four de novo mutational signatures, one of which matches the COSMIC APOBEC-associated signature 2. The viral genomes of the precancer/cancer cases are less likely to contain within-host somatic HPV16 APOBEC3-induced mutations (Fisher’s exact test, *P* = 6.2 x 10^−14^), and have a 30% lower nonsynonymous APOBEC3 mutation burden compared to controls. We replicate the low prevalence of HPV16 APOBEC3-induced mutations in 1,749 additional cases. APOBEC3 mutations also historically contribute to the evolution of HPV16 lineages. We demonstrate that cervical infections with a greater burden of somatic HPV16 APOBEC3-induced mutations are more likely to be benign or subsequently clear, suggesting they may reduce persistence, and thus progression, within the host.

## Abstract (web summary)

The APOBEC mutational signature is prevalent in different tumour types. Here, using HPV16- positive cervical samples, the authors show that the signature is more prevalent in the viral genome of benign or clearing HPV16 infections compared to the viral genomes of the more advanced precancerous lesions or cervical cancer.

## Introduction

High-risk human papillomaviruses (HR-HPVs) are small double-stranded DNA viruses that cause cervical cancer^1^ and a large proportion of other anogenital and oropharyngeal cancers^2,3^. HPV16 is the most potent of the 12 HR-HPV types; it accounts for >50% of the >500,000 incident cervical cancer cases worldwide annually^4–6^. It is unknown why HPV16 is more carcinogenic than other HPV types, or why the majority of HPV16 infections “clear” (are either eliminated or controlled) while others persist and lead to cervical precancer and cancer^7–9^. Within HPV16, genetic variation partly predicts risk of precancer and cancer. For example, the sublineages (A1–A4, B1–B4, C1–C4, D1–D4) of HPV16, defined by genetic variation, have been associated with substantial differences in cervical carcinogenicity^10–22^, and specific sublineages are linked to adenocarcinomas with an odds ratio (OR) of >100^21^. There is also much finer genetic variation among circulating HPV16 isolates^23,24^, which varies by viral gene region and infection outcome, with more variation observed in women with low-grade or benign HPV16 infections than in those with cancers^24,25^. Most notably, the *E7* oncogene lacks nonsynonymous (amino acid changing) variants in cervical cancers from around the world compared with controls, illustrating that rigid *E7* conservation is necessary for carcinogenicity^24^.

While cell-mediated immunity is thought to explain much of HPV clearance, innate immunity may also be important^26^. Specifically, the expression of human apolipoprotein B mRNA-editing, enzyme-catalytic, polypeptide-like 3 (APOBEC3) family of cytidine deaminases, APOBEC3A or APOBEC3B (hA3A/B), have been shown to be upregulated following HPV infection and act as a restriction factor, activated by the HPV16 E6 and E7 oncoproteins^27–32^. This elevated activity of hA3A/B enzymes can mediate mutations in both the host and viral genomes^32–34^. Large comprehensive human cancer genomic studies have characterized somatic mutations and discovered APOBEC3-mediated mutational signatures among multiple cancer types^35–41^, particularly enriched in HPV-positive cervical^35^ and HPV-positive head and neck cancers^42–44^. A recent study of oral squamous cell carcinomas (OSCCs) determined that the somatic APOBEC3 mutation burden was strongly linked to the total mutation burden in HPV-positive, but not HPV-negative OSCCs^44^. APOBEC3 mutagenesis is a source of oncogenic driver events^35,43–45^ (e.g., *PIK3CA* E545K hotspot mutations in cervical cancers^35^) and contributes to clonal evolution and intratumor heterogeneity^46^ (for more details, see refs. ^32,47–49^). At the same time, APOBEC3 cytidine deaminases have been shown to induce mutations in HPV genomes and act as a restriction factor in the early stages of HPV infection^30,50,51^.

A targeted sequencing study of 9 HPV16 precancers demonstrated that the HPV16 genome was hyperedited with G > A and C > T changes by human APOBEC3 cytidine deaminases^52^; subsequently, G > A and C > T hypermutations were verified in two small studies of the *E2* gene and long control region (LCR) of the HPV16 genome^53,54^. A recent study showed that APOBEC3 cytidine deaminase was a driver of HPV mutations at the trinucleotide APOBEC3 target, TpCpN, across 151 HPV16/52/58 whole genomes^25^, particularly in low-grade lesions. It appears that host–pathogen coevolution has selected for HPV16 genomes with fewer APOBEC3 attackable motifs^55^. Thus, the remaining motifs are probably necessary or important for the full infectious viral life cycle. There have been no large-scale genomic studies to comprehensively characterize APOBEC3 mutagenesis and other mutational signatures across the HPV16 genome in cervical precancer/cancer cases and controls.

To investigate the viral genetic variation across the HPV16 genome potentially induced by human APOBEC3 cytidine deaminases and other mutational processes (signatures) and evaluate how these variants contribute to infection outcome (i.e., viral clearance or carcinogenesis), we analyze HPV16 whole-genome sequence data from 5328 case–control samples. Our primary analysis includes 3579 HPV16 genomes^21,24^ from 1265 controls (women with a benign HPV16 infection defined as causing ≤ cervical intraepithelial neoplasia [CIN] grade 1 [CIN1], and/or “clearing”) and 1032 CIN2, 1170 CIN3 precancer, and 112 cancer cases in the prospective NCI-Kaiser Permanente Northern California (PaP) Cohort^56^. We replicate case findings in 1749 HPV16 genomes^24^, including HPV16 from 444 CIN3 precancer and cancer cases (i.e., CIN3+) in a cross-sectional U.S. population^17,57–59^ from the Study to Understand Cervical Cancer Early Endpoints and Determinants (SUCCEED), and 1305 invasive cervical cancers collected internationally by the International Agency for Research on Cancer (IARC)^60–63^.

## Results

### Whole-genome sequencing of HPV16

Using a PCR based next-generation sequencing (NGS) assay^64^, we performed HPV16 whole-genome sequencing of 5328 HPV16-positive cervical precancer/cancer cases and controls (Table 1). The mean number of sequencing reads aligned across the HPV16 genome per sample was 520,127 (standard error 76,629), with a mean of 3696 sequencing reads per gene region.

**Table 1:** Summary of HPV16-infected women from three studies by case status.

Study	Status	No. of women
NCI-Kaiser PaP	Control	1265
	CIN2	1032
	CIN3	1170
	Cancer	112
SUCCEED	CIN3	314
	Cancer	130
IARC	Cancer	1305
Total		5328

*CIN2* cervical intraepithelial neoplasia (CIN) grade 2, *CIN3* CIN grade 3.

### Mutational signature analysis identified four signatures

**Fig. 1:**
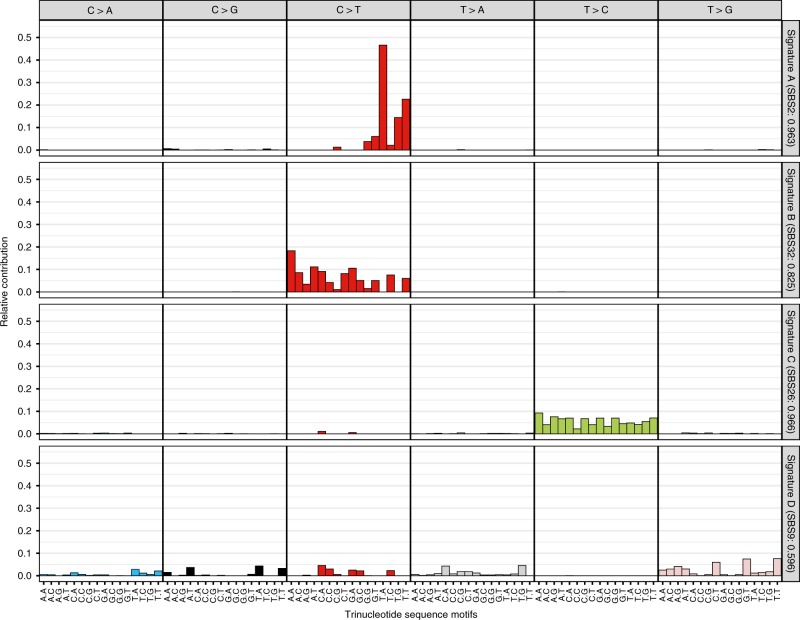
Four de novo mutational signatures identified using all variants across the HPV16 genome in women from the PaP cohort. The *x*-axis indicates the 5′ and 3′ nucleotides for each of the top panel substitutions for the three base-pair motifs. The *y*-axis shows the single-base substitution (SBS) composition of each mutational signature by the 96 trinucleotide sequence motifs. For each identified signature, shown as A–D, the similarity was determined to the known COSMIC SBS signatures (https://cancer.sanger.ac.uk/cosmic/signatures/SBS/). The identified signature letter (A–D) and in parentheses the most similar COSMIC SBS signature number along with the cosine similarity are shown along the right *y*-axis. Cosine similarity ranges from 0 to 1, with a cosine of 1 indicating a perfect match.

We first characterized the 96 trinucleotide mutation types taking into account the sequence context immediately 5′ and 3′ to each mutated base, and conducted a de novo mutational signature analysis of all variants across 3579 HPV16 genomes. We identified four mutational signatures present in the HPV16 genomes (Fig. 1). Signature A was characterized by C > T mutations enriched at the TCW motif, and it was highly similar to the known COSMIC (the Catalogue of Somatic Mutations in Cancer)^65^ single-base substitution (SBS) signature 2 (cosine similarity = 0.963), which has been associated with the activity of the APOBEC cytidine deaminases. Signature B was represented by C > T mutations outside the TCW motif (excluding C > T mutations in signature A), with some similarity to COSMIC^65^ SBS signature 32 (cosine similarity = 0.825), the etiology of this signature is less understood and suggested to be associated with damage to guanine and repair by transcription-coupled nucleotide excision repair. Signature C was characterized by T > C mutations, and highly similar to COSMIC^65^ SBS signature 26 (cosine similarity = 0.966), which has been associated with defective mismatch repair. The fourth signature D illustrated a relatively flat signature across many different mutation types with little similarity to a known COSMIC^65^ SBS signature.

We further evaluated the distribution of the mutations identified by each signature in our case–control samples and examined if they were linked to viral clearance or carcinogenicity with a primary focus on the HPV16 APOBEC3-induced mutations.

### HPV16 APOBEC3 mutations are more prominent at a low variant allele fraction

**Fig. 2:**
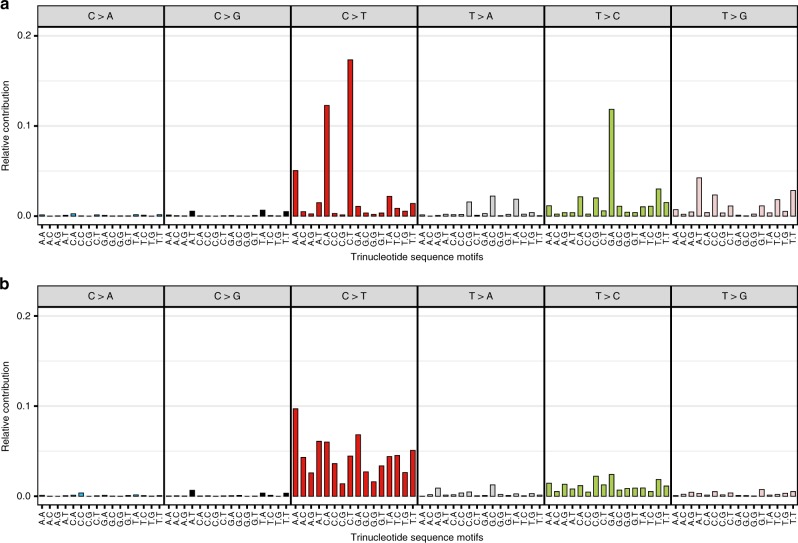
Frequency of the 96 trinucleotide mutation types for variants across the HPV16 genome in women from the PaP cohort. Illustrated for (**a**) high variant allele fraction (VAF) and (**b**) low VAF variants. The *x*-axis indicates the 5′ and 3′ nucleotides for each of the top panel substitutions for the three base-pair motifs.

As expected, cytosine-to-thymine (C > T) changes were the most frequent variants observed (48.7% of total mutations; Supplementary Fig. 1). We observed that the distribution of mutation types differed by variant allele fraction (VAF; Fig. 2a, b). VAF refers to the fraction or percent of total viral sequence reads per woman containing the mutation at a given genomic position (see “Methods” for more details). The VAF is expected to be ~1.0 if the variant was present in the virus at acquisition (i.e., constitutive variant in a haploid HPV genome), and in a lower fraction of the reads if the viral mutation occurred de novo during the infection period (i.e., a within-host somatic mutation). We evaluated constitutive HPV16 variants at a high VAF in each woman, defined based on the distribution of mutations as a variant occurring in >60% of the sequence reads at that locus, and within-host HPV16 somatic mutations at a low VAF, defined as occurring in 10–60% of the sequence reads (variants in <10% of the reads were excluded), separately.

The C > T substitutions were most prominent at low VAFs (Fig. 2a, b). The previously established APOBEC3 mutation types^39^, characterized by a C > T substitution with thymine on its 5′ side and adenine or thymine on its 3′ side (TCW motif [W is A or T]), identified as mutational signature A, and also C > T substitutions outside the TCW motif (signature B), were specifically more prevalent at a low VAF compared with high VAF (APOBEC3 mutations: 10.2% of low VAF, and 4.8% of high VAF; proportion test, *P*-value < 0.001; C > T outside the TCW motif: 59.8% of low VAF, and 40.7% of high VAF; proportion test, *P*-value < 0.001). However, APOBEC3-associated^39^ C > G substitutions at the TCW motif were rare across the HPV16 genome at both low and high VAFs (0.7% of low VAF, and 1.2% of high VAF). Supplementary Fig. 2 illustrates the distribution of APOBEC3-induced mutations across levels of VAF and shows that most mutations were in either very low or very high VAF levels. The within-host viral somatic mutations at low VAF were most pronounced in the controls, while the highest VAF constitutive variants, likely representing variants present in the HPV16 genome at acquisition, were equivalent among cases and controls.

We also observed mutation types characterized by T > C (signature C) and T > G (signature D) mutations across the HPV16 genomes at high VAF (Fig. 2a). T > G mutations are expected to be rare DNA changes^66^; the reason for their higher frequency in HPV16 genomes is unknown.

### Within-host HPV16 APOBEC3 mutations are more frequent in controls

**Fig. 3:**
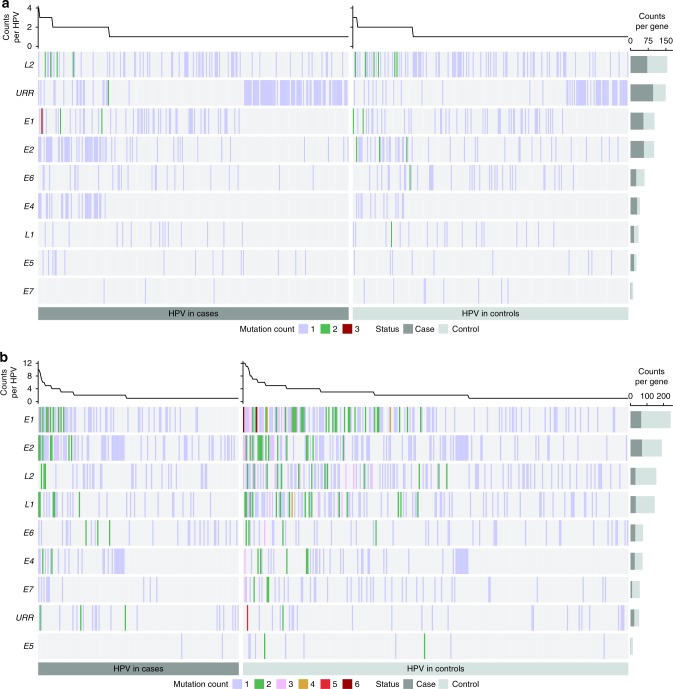
The number of APOBEC3-induced mutations across the HPV16 genome by gene region in women from the PaP cohort. The plots show only the cases and controls with one or more APOBEC3-induced mutations at a (**a**) high variant allele fraction (VAF) and (**b**) low VAF. Each vertical line represents a sample with at least one APOBEC3-induced mutation, colored by the number of mutations observed, as 1–3 (see legend), per viral gene region. Samples with no APOBEC3-induced mutations are not illustrated; the size of the case and control panels correspond to the number of individuals with at least one APOBEC3-induced mutation. The samples are organized along the *x*-axis by status (case vs. control). Cases are cervical intraepithelial neoplasia grade 3 and cancer cases (CIN3+). The right *y*-axis represents viral gene regions with the overall frequency of APOBEC3 mutations summarized, taking into account the sample sizes of the cases/controls and potential APOBEC3-mutable sites, for CIN3+ cases in dark gray and controls in light gray. The top panel histogram summarizes the total APOBEC3-induced mutations for the cases and controls across the HPV16 genomes. URR upstream regulatory region, *E6* early gene 6, *E7* early gene 7, *E1* early gene 1, *E2* early gene 2, *E4*  early gene 4, *E5*  early gene 5, *L2*  late gene 2, *L1*  late gene 1.

To test if APOBEC3-induced mutations, and the other mutational signature substitutions, were associated with case–control status, we compared the HPV16 APOBEC3-induced mutations (Fig. 3a, b) in the precancer/cancer cases and controls stratified by low/high VAF (see “Methods” for more details). Within-host viral somatic APOBEC3-induced mutations were present in significantly fewer CIN3 + cases (11.9%) compared with controls (23.2%; OR 0.45, 95% CI 0.36–0.56, Fisher’s exact test, *P*-value = 6.2 × 10^−14^; Table 2). The results were similar for CIN2 + cases compared with controls (OR 0.48, 95% CI 0.40–0.57, Fisher’s exact test, *P*-value = 5.8 × 10^−16^; Supplementary Table 1). In a subset analysis, we compared only the incident cases (*N* = 333) that developed CIN3 + during the follow-up study period (i.e., after baseline or enrollment) to the controls (i.e., women that cleared their HPV16 or did not progress to CIN2+), and also showed that the incident CIN3+ cases had significantly fewer somatic APOBEC3-induced mutations than controls (OR 0.57, 95% CI 0.41–0.79, Fisher’s exact test, *P*-value = 0.0003). We further evaluated if somatic APOBEC3-induced mutations were associated with case/control status for each viral gene region. *L1* and *L2* gene regions had significantly more somatic APOBEC3-induced nonsynonymous mutations in the controls compared with CIN3+ cases (Wald test, *P*-value = 0.01 and 6.7 × 10^−4^, respectively; Supplementary Table 2). There was no apparent clustering of mutations in these gene regions by functional domain (Supplementary Fig. 3a, b).

**Table 2:** Cases and controls with and without APOBEC3-induced mutations by variant allele fraction in the NCI-Kaiser PaP cohort.

VAF	Status	No APOBEC3 mutation	APOBEC3 mutation	%	OR	95% CI	*P*-value^a^
Low^b^	CIN3+	1129	153	11.9%	0.45	(0.36–0.56)	6.2 × 10^−14^
	Control	971	294	23.2%	Ref.		
High^c^	CIN3 +	997	285	22.2%	1.14	(0.94–1.39)	0.17
	Control	1012	253	20.0%	Ref.		

*VAF* variant allele fraction, *CIN3+* cervical intraepithelial neoplasia grade 3 and cancer cases, *OR* odds ratio, *CI* confidence intervals, *Ref*. referent group.

^a^Fisher’s exact test, two-sided, comparing the number of women with at least one APOBEC3-induced mutation and those without APOBEC3-induced mutations in the CIN3+ cases to controls.

^b^Low VAF is defined as VAF >10% and < = 60%.

^c^High VAF is defined as VAF >60%.

In contrast, there was no significant difference between the high VAF, constitutive HPV16 APOBEC3-induced variants in the CIN3+ cases compared with controls (OR 1.14, 95% CI 0.94–1.39, Fisher’s exact test, *P*-value = 0.17; Table 2). By gene region, there were more constitutive APOBEC3 nonsynonymous variants in the cases compared with controls in *E4* (Mutation burden ratio 7.99, 95% CI 1.85–34.4; Wald test, *P*-value = 0.01; Supplementary Table 2).

We replicated the prevalence of APOBEC3-induced mutations throughout the HPV16 genome in precancer/cancer cases in two independent case study populations at both low and high VAFs (Supplementary Table 3). In particular, 12.4% and 12.5% of precancer/cancer cases had HPV16 somatic APOBEC3-induced mutations in SUCCEED and IARC, respectively, similar to the 11.9% observed in the PaP cohort cases and significantly fewer than the PaP controls (23.2%; OR 0.47, Fisher’s exact test, *P*-value = 5.1 × 10^−7^ and 1.1 × 10^−12^, respectively). The frequency of HPV16 APOBEC3-induced variants at high VAF among the SUCCEED and IARC CIN3 + cases was similar to the PaP CIN3 + cases, and slightly higher than the PaP controls (Supplementary Table 3).

Within-host viral somatic C > T substitutions outside the TCW motif (signature B) and the T > C substitutions (signature C) were also present in significantly fewer CIN3 + cases (36.1% and 18.5%, respectively) compared with controls (43.2% and 32.9%, respectively; Fisher’s exact test *P*-value < 0.001) in the PaP cohort (Supplementary Table 4). However, the frequency of these specific substitutions did not replicate in the precancer/cancer cases in our two independent case study populations (SUCCEED and IARC), so we did not evaluate their relationship to case–control status further. The substitutions characterized by mutational signature D were not associated with case–control status at low VAF (Supplementary Table 4).

### Characteristics of HPV16 mutations induced by APOBEC3 enzymes

We first estimated all possible DNA changes and resultant amino acid changes across the HPV16 reference genome. For the HPV16 A1 sublineage reference genome (see Methods), there were a total of 263 APOBEC3 targetable sites, and fewer targetable sites for the more carcinogenic HPV16 D2/D3 sublineage^10–22^ genomes, 247/246 APOBEC3 targetable sites (Supplementary Table 5). Although HPV16 A1/A2 sublineages had only 14–17 more APOBEC3 targetable sites than the D2/D3 sublineages, the controls with A1/A2 sublineages had more APOBEC3 mutations than D2/D3 (31.2% vs. 15.8%; *P* = 0.04). Of the possible HPV16 APOBEC3 targetable sites (A1 reference genome), 96.2% of these APOBEC3 substitutions would result in a nonsynonymous change compared with 77.2% of non-APOBEC3 sites (proportion test, *P*-value < 0.001). Consequently, 95.2% of the APOBEC3-induced mutations we observed were nonsynonymous, which was significantly higher than the proportion observed for the non-APOBEC3 mutations, 71.2% (proportion test, *P*-value < 0.001**;** Supplementary Table 6).

For the HPV16 infections with at least one APOBEC3-induced mutation, we compared the APOBEC3-induced mutation burden per virus in cases vs. controls and between nonsynonymous vs. synonymous mutations, adjusting for the number of possible HPV16 APOBEC3-mutable sites (see “Methods” for more details). For within-host viral somatic mutations, CIN3 + cases had a significantly lower APOBEC3-induced mutation burden compared with controls (mutation burden ratio 0.71, 95% CI 0.61–0.82; Wald test, *P*-value = 6.42 × 10^−6^) for all mutations and stratified by both nonsynonymous (mutation burden ratio 0.71; Wald test, *P*-value = 1.2 × 10^−5^) and synonymous mutations (ratio 0.68; Wald test, *P*-value = 0.28) (Table 3; Supplementary Table 7). There was no evidence of selection against nonsynonymous relative to synonymous mutations (ratio of nonsynonymous-to-synonymous mutation rate 1.0; Wald test, *P*-value = 0.99; Table 3). Here, the nonsynonymous and synonymous mutation rates were evaluated based on the number expected relative to the number of APOBEC3 targetable sites that could result in a nonsynonymous or synonymous mutation. Most of these somatic HPV16 APOBEC3-induced mutations at low VAF were rare in the population with a minor allele frequency (MAF) < 0.01, and rare somatic APOBEC3 mutations were significantly more frequent in the controls compared to the cases (proportion test, *P*-value = 1.5 × 10^−4^).

**Table 3:** Burden of APOBEC3-induced mutations in cases and controls from the NCI-Kaiser PaP cohort. Mutations are compared in CIN3+ cases vs. controls and for nonsynonymous (nonsyn) vs. synonymous (syn) mutations by variant allele fraction.

VAF	Parameter	Interpretation	Mutation burden^a^	95% CI	*P*-value^d^
Low^b^	*r*_syn_	Enrichment of synonymous mutations in cases vs. controls	0.68	(0.34–1.36)	0.28
	*r*_nonsyn_	Enrichment of nonsynonymous mutations in cases vs. controls	0.71	(0.61–0.83)	1.2 × 10^−5^
	W	Selection of nonsynonymous mutations vs. synonymous mutations in controls	1.00	(0.70–1.44)	0.99
High^c^	*r*_syn_	Enrichment of synonymous mutations in cases vs. controls	1.27	(0.73–2.21)	0.40
	*r*_nonsyn_	Enrichment of nonsynonymous mutations in cases vs. controls	0.82	(0.68–1.00)	0.05
	W	Selection of nonsynonymous mutations vs. synonymous mutations in controls	0.55	(0.35–0.85)	7.9 × 10^−3^

*VAF* variant allele fraction, *CIN3+* cervical intraepithelial neoplasia grade 3 and cancer cases, *CI* confidence intervals.

^a^Mutation burden ratio of APOBEC3-induced mutations was calculated using a Poisson regression model to compare the mutation burden or enrichment of APOBEC3-induced mutations per virus between cases and controls for nonsynonymous and synonymous mutations (*r*); selection of nonsynonymous mutations in the controls was estimated adjusting for the number of cases and controls and the potential APOBEC3-mutable bases that result in nonsynonymous and synonymous mutations (w).

^b^Low VAF is defined as VAF >10% and < = 60%.

^c^High VAF is defined as VAF >60%.

^d^*P*-values are generated by the Wald test of a Poisson regression model.

In contrast, at high VAF, there was no significant difference in the mutation burden of APOBEC3-induced nonsynonymous and synonymous mutations in cases and controls, and there was evidence of negative selection against nonsynonymous mutations relative to synonymous mutations (mutation burden ratio 0.55; Wald test, *P*-value = 7.9 × 10^−3^; Table 3).

Among the possible APOBEC3 targetable bases on each strand, the percent mutated on the positive strand (5.8/kb) was comparable with that estimated on the negative strand (6.2/kb, proportion test, *P*-value = 0.61; Supplementary Table 8). In addition, among the APOBEC3A (YTCA, Y is a pyrimidine base) and APOBEC3B (RTCA, R is a purine base) possible mutable bases^67^, we compared the rate of having at least one APOEBC3A or APOBEC3B mutation and found no significant difference (0.35 vs. 0.34/kb, proportion test, *P*-value = 0.86; Supplementary Table 9). When further separating APOBEC3-induced mutations by high/low VAF, there was no significant difference between the APOBEC3 mutation rates on the positive or negative strand, or for APOBEC3A or APOBEC3B mutations (Supplementary Tables 8 and 9).

### APOBEC mutations contributed to the evolution of HPV16 lineages

We determined that APOBEC3 editing may have contributed to the evolution of HPV16 lineages using 239 HPV16 non-A1/2 sublineage sequences. We specifically evaluated the HPV16 nucleotide positions that are known to “define” each of the HPV16 main lineages^68^ (i.e., lineage/sublineage diagnostic SNPs; each SNP in the lineage haplotypes) compared with the derived ancestral sequence for each main lineage and sublineage at each node of the phylogenetic tree (Supplementary Fig. 4). We determined that there was a range of 6–41% of the lineage-defining SNPs for each of the different sublineages potentially induced by APOBEC3 (Supplementary Fig. 4). The D2/D3 sublineages, which are known to be the most carcinogenic of the HPV16 sublineages^21^, had the greatest number of lineage-defining SNPs potentially induced by APOBEC3 (35 and 41%, respectively).

## Discussion

We report the largest HPV16 whole-genome sequencing study to date evaluating viral genome mutational signatures and identify viral APOBEC3 mutations likely induced during a woman’s infection linked to benign or clearing infections. We^24^ and others^23^ have previously shown that there is high genetic diversity among HPV16 isolates circulating in the population; these studies were focused on viral SNPs presumed to be “inherited” variants present at HPV acquisition and detected in all or nearly all viral sequence reads at a given locus (high VAF). In contrast, here we use deep NGS to more finely evaluate “acquired” somatic mutations presumed to be induced recently during a woman’s infection and detected in a minority of sequence reads (low VAF, but with sufficient read depth >10×). We have discovered additional somatic viral genetic diversity that is likely driven by APOBEC3 activity and associated with benign infections or subsequent viral clearance in our large prospective cohort. Our data suggest that these APOBEC3-induced mutations may constrain the viability, and by extension the oncogenic potential, of HPV16. We further determined that mutations induced by APOBEC3 cytidine deaminases contributed to HPV16 genetic diversity that shaped viral evolution of the important HPV16 lineages.

The combination of our large study population and deep NGS technology has revealed that HPV16 genomes, which have generally been considered stable during a persistent infection, can accumulate somatic mutations during infection, presenting in a fraction of the viruses within a host driven by APOBEC3 activity and other mutational processes. These somatic mutations, including APOBEC3-induced mutations, likely result in clearance or a reduced ability of the virus to persist. We observed four mutational signatures across HPV16 genomes, and the somatic substitutions related to the three main mutational signatures (A, B, and C) were all more prevalent in the controls compared with the precancer/cancer cases. However, the frequency of somatic substitutions related to signatures B and C did not replicate in two additional case populations; further follow-up is needed to determine the etiology and relevance of these substitutions. The APOBEC3-induced mutations (signature A) were enriched in the controls compared with cases in all three of our precancer/cancer case populations. This suggests that APOBEC3 is primarily inducing mutations during a woman’s infection when HPV16 is replicating, and ssDNA is exposed and targetable, during a productive infection^69^ in benign or low-grade (<CIN2) lesions. This APOBEC3 mutagenesis within the host prior to HPV clearance and transmission likely additionally contributes to the high diversity of HPV16 in the population previously reported^23,24^, and to viral evolution^25,55,70^.

There was no disparity between the mutation burden of nonsynonymous (i.e., missense or nonsense) and synonymous somatic mutations in controls, suggesting that these mutations are likely recent somatic mutations arising during a woman’s infection which would not have had time to be selectively removed from the viral population (i.e., ineffective purifying selection). These mutations were also most often rare in our population (i.e., observed in <1% of women) or singletons (i.e., observed in only one woman among 3500+), further suggesting that most of them arose recently within the host. It is possible that a minority of the rare low VAF variants could be due to PCR error or artifacts, although given our stringent quality control including the requirement that all variants be present in >10 sequence reads, our low assay error rate, and the specific enrichment of APOBEC3 signature mutations in controls at a low VAF instead of random mutations across all samples (as would be expected from errors), this is likely minimal. We observed a significantly higher proportion of viral nonsynonymous APOBEC3-induced mutations compared with that for other non-APOBEC3 mutations. This is consistent with the TpC dinucleotide depletion at the third codon position observed in the viral open-reading frame^55,70^, which would result in our observed viral APOBEC3-induced mutations primarily occurring at the first and second codon positions and thus causing the enrichment of nonsynonymous changes. Given that 95% of the APOBEC3-induced mutations were nonsynonymous and more frequent in the controls, we presume these mutations were deleterious to viral persistence and thus constrained the viability of HPV16. Alternatively, it is also possible that these APOBEC3-induced mutations are a biomarker of an innate immune response to the virus.

Earlier targeted HPV16 sequencing studies^52–54^ and an HPV whole-genome sequencing study of 151 HPV16, HPV52, or HPV58 samples^25^ also identify APOBEC3-induced mutations in HPV genomes. Our within-host viral somatic mutations are consistent with the study by Hirose et al.^25^, suggesting that the high levels of HPV genomic variation they observed, particularly in the low-grade CIN1 lesions, were likely the result of accumulating somatic mutations during infection. For a more direct comparison of our data with the HPV16 genome data from Hirose et al.^25^, we downloaded their 45 HPV16 genomes (GenBank accession numbers: LC368952 to LC368996) and created a 96 trinucleotide mutation-type plot after exclusion of the common evolutionary-derived HPV16 lineage-defining substitutions^68,71^, since inclusion of lineage-defining substitutions would skew the distribution for evaluations of recent or within-host mutations. The resulting distribution of mutation types looks similar to ours (Supplementary Fig. 5), including specific APOBEC3-associated variants and T > C and T > G substitutions. Although, mutational signature extraction was not possible for these 45 genomes^25^ due to the overall small number of variants.

Interestingly, we did not detect leading/lagging or transcribed DNA strand biases or APOBEC3-associated C > G changes at TCW motifs, which correspond to the APOBEC3 COSMIC^65^ SBS signature 13, as observed in human somatic genomes related to APOBEC mutagenesis^36,39^. Our observations are consistent with previous HPV data^25,52^ and suggest a difference between HPV and human genome APOBEC3 mutagenesis. It is possible that the antiviral APOBEC3 response to HPV infection is separate or slightly different from the role of APOBEC3 in host somatic genome mutagenesis.

The distribution of APOBEC3-induced mutations across the viral genome was different between the controls and precancer/cancer cases, suggesting that sites in specific regions of the viral genome may be “hit” more often by APOBEC3, or mutations at specific sites may have more deleterious effects to the virus if absent from the cases, such as mutations in *L1* and *L2*. Alternatively, since APOBEC3 mutations occur when the viral DNA is single stranded, the mutations observed in the *L1* and *L2* gene regions more frequently in the controls may reflect that these regions of the viral genome are more likely to be transcribed and single stranded in the controls with a productive infection.

As noted, HPVs have evolved to limit the number of TpC dinucleotides in their genomes to avoid restriction^55,70^, yet even with a limited number of APOBEC3 targetable sites, somatic APOBEC3-induced mutations were still observed and enriched in benign infections, suggesting an antiviral effect. We note that the majority of viruses did not have APOBEC3-induced mutations. We may be underestimating the level of APOBEC3-induced mutations due to our stringent quality control and low VAF cut point of 10%, instead of >0.5%^25^, which would not detect the lowest VAF-induced mutations. It is also possible that some of the viruses with somatic APOBEC3-induced mutations were rapidly cleared, if they were less viable, and thus not part of our study.

In contrast, the high VAF constitutive APOBEC3 variants, which included a lower burden of nonsynonymous variants, were likely present in the HPV16 population for a longer period of time and/or present at viral acquisition and reflect the natural variants circulating in the population. Purifying selection would have prevented a disproportionate number of nonsynonymous variants from reaching high frequencies in HPV16 populations, since they are more likely than synonymous variants to be disadvantageous (e.g., leading to a reduced ability to persist in the host), as we observed. These high VAF APOBEC3 variants were overall equivalent in cases and controls, and possibly neutral with respect to carcinogenesis. However, we did observe that there were specifically more high VAF *E4* variants in the cases, suggesting that these genetic variants could be slightly advantageous to viral persistence.

APOBEC3 upregulation occurs throughout disease progression to inhibit the HPV infection^30^. However, we still observed absent or reduced somatic APOBEC3-induced mutations in the precancer/cancer cases, which represent infections that have been successfully persisting for years, suggesting that these viruses may be evading host restriction by APOBEC3 and/or the more homogeneous viral genomes in the cases reflects outgrowth of a clone with a selective growth advantage. The somatic viral APOBEC3 mutations induced within the host that are not deleterious to the virus may instead aid in evasion of the host adaptive immune response by altering viral antigens, and this viral clone would then be selected for in that host. It is also possible that the precancer/cancer HPV16 viruses may be partially evading restriction through integration of HPV DNA into the host genome, where a portion of the viral genome and episomal HPV genomes are lost^69,72^ and therefore there is less viral DNA present in these infections to be targeted by APOBEC3 enzymes. In addition, increased viral DNA methylation of the cases^73,74^ may partially protect the viral genome from mutation by APOBEC3 enzymes. If APOBEC3 activity is upregulated and failed to clear HPV in the cases with advanced lesions, this may be contributing to the off-target host somatic mutations observed in cervical and other HPV-associated cancers. The difference in APOBEC3-induced mutations in the cases and controls may also reflect differences in activation and/or regulation of the innate immune response that leads to APOBEC3 expression and downstream viral mutations^32^, partially related to the activity of IFN-α^75,76^, inflammation and NF-κB signaling^77,78^, *TP53*^31,79^, or human genetic variation^44,80,81^.

**Fig. 4:**
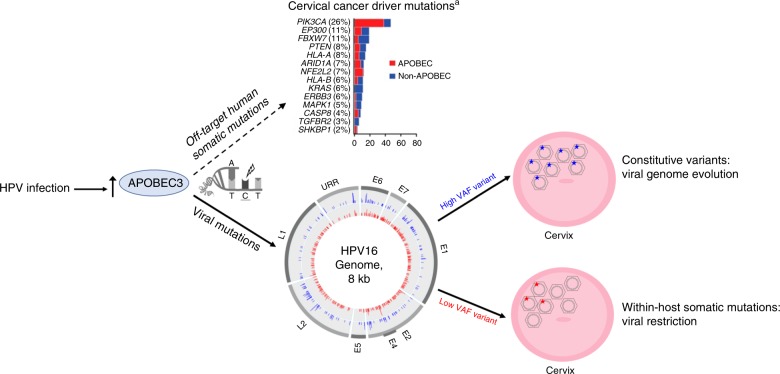
Summary of the effects of mutations induced by the activity of APOBEC3. Viral APOBEC3-induced mutations are illustrated in the circle plot by viral gene region in the inner ring for low variant allele fraction (VAF) somatic mutations in red, and the outer ring for high VAF constitutive variants in blue for all individuals in the PaP cohort (mutations in both cases and controls are illustrated). Modified from the Cancer Genome Atlas Research Network^35^.

In summary (Fig. 4), we determined that APOBEC3 cytidine deaminases induce somatic mutations across the HPV16 genome, these mutations have contributed to the evolution of important viral lineages, and infections with somatic viral mutations induced during a woman’s infection were more likely to become benign infections or infections that subsequently cleared.

## Methods

### Study populations

The cases and controls for the large discovery phase of our study were chosen from the Kaiser Permanente Northern California (KPNC)-NCI HPV Persistence and Progression (PaP) cohort^21^. This study population has been previously described^56^. The HPV Persistence and Progression (PaP) Cohort is a repository of residual cervical specimens stored in specimen transport medium (STM; Qiagen, Valencia, CA), from women who underwent cervical cancer screening from January 2007 to January 2011 at Kaiser Permanente Northern California (KPNC). Women could opt out of having their residual cervical specimens retained; only 8% of women with collected specimens opted out from having their specimen banked and tested. De-identified demographic and clinical information as well as all HPV and cytology test results and cervical histopathology were obtained on the cohort from electronic health records.

This cross-sectional study included 3579 exfoliated cervical cell specimens collected at enrollment previously determined to contain HPV16 DNA^56^, including 112 cancers, 1170 CIN3, 1032 CIN2, and 1265 controls (<CIN2) in follow-up through 2015. The precancer (CIN2 and CIN3) and cancer cases were diagnosed at baseline (i.e., at enrollment; prevalent cases) or during the study follow-up period after the baseline specimens were collected (i.e., incident cases). The controls were defined as women having enrollment specimens with HPV16 DNA, and no histologic evidence of equivocal precancer or worse (CIN2+) during the follow-up study period according to the coded data obtained from electronic health records. Therefore, controls were the HPV16-positive women that either cleared their infections or had not progressed to CIN2+ during the follow-up study period. Women are followed as long as possible, and only censored if they received treatment for a CIN2+ lesion, or until the last documented follow-up cytology or histology. The study protocol was reviewed and approved yearly by Kaiser Permanente and the National Cancer Institute Institutional Review Boards.

To confirm the findings from the PaP Cohort, we evaluated HPV16-positive women from the Study to Understand Cervical Cancer Early Endpoints and Determinants (SUCCEED). The details of the study design and specimen collection were previously described^57–59^. Briefly, a total of 2004 women were enrolled into SUCCEED between November 2003 and October 2009. We recruited women that were referred to colposcopy or treatment at the University of Oklahoma Dysplasia Clinic based at the University of Oklahoma Health Sciences Centre (OUHSC), with a recent abnormal Pap smear diagnosis or a biopsy diagnosis of CIN/cancer. Here, we included all CIN3+ exfoliated cervical cell specimens previously found to contain HPV16 DNA, including 444 women: 314 CIN3 and 130 cancer cases. Written informed consent was obtained from all women enrolled in the study, and Institutional Review Board approval was provided by OUHSC and the US National Cancer Institute.

In total, 645 additional HPV16-positive cervical cell or tissue (frozen biopsy or formalin-fixed paraffin-embedded [FFPE]) specimens from cervical cancer cases were studied to assess the worldwide generalizability of our main finding, from the biobank at IARC. These samples were part of the IARC-coordinated cervical cancer case series, cervical cancer case–control studies and population-based HPV prevalence surveys from 39 countries worldwide^60–63^. Both local and IARC ethical committees approved all studies. We sequenced all HPV16-positive histologically confirmed cervical cancers with adequate DNA left in the IARC biobank.

### HPV16 detection and DNA isolation

DNA was extracted from the banked STM specimens as previously described^82^. Typing methods varied for different subsets of the cohort, many of the enrollment PaP samples were tested by the Burk laboratory (Bronx, NY) using MY09/M11 *L1* degenerate primer PCR (MY09/11 PCR) and type-specific dot-blot hybridization methods^82,83^. Other specimens were tested with either the Linear Array^®^ HPV Genotyping System (Roche Molecular Diagnostics, Pleasanton, CA) or typed by BD using Onclarity (BD, Sparks, MD).

Details of DNA isolation and HPV detection have been previously described^57,84^. Briefly, DNA was isolated from 1 mL aliquots of PreservCyt-fixed cells using the QIAamp DNA Blood Mini Kit (Qiagen) following a rinse in Hanks’ balanced salt solution (HBSS). The Linear Array^®^ HPV Genotyping System (Roche Molecular Diagnostics) was used to detect HPV genotypes. Hybridization of PCR products to linear arrays and subsequent signal detection were performed using the Auto-LiPA automated staining system (Innogenetics N.V., Belgium). Hybridization to both β-globin probes was required to report genotyping results. A hybridization signal was called “positive” when an unambiguous, continuous band was observed on the array.

DNA was extracted from frozen biopsy specimens, cervical cells, or FFPE at IARC, as previously described^85^. Samples were genotyped for 37 HPV types using a GP5+/6+-based PCR system^86^ in one centralized laboratory (Department of Molecular Pathology, Vrije University, Amsterdam, The Netherlands).

### Ion Torrent library preparation and sequencing

We used a custom Thermo Fisher Ion Torrent AmpliSeq HPV16 panel approach to amplify the entire 7906 bp HPV16 genome, as previously described^64^. In brief, the next-generation sequencing (NGS) assay used the Thermo Fisher Life Sciences’ Ion Torrent S5 and a custom HPV16 Ion Ampliseq panel of 47 multiplexed primer sets. Custom overlapping degenerate primers were designed to cover the entire viral genomes for all HPV16 variant lineages. After amplification, an Ion Torrent adapter-ligated library was generated following the manufacturer’s Ion AmpliSeq Library Preparation kit 2.0-96LV protocol with slight modifications (Life Technologies, Part #4480441). Raw sequencing reads were quality and adaptor trimmed using the Torrent Suite™ Software and aligned to the HPV16 reference sequence (7906 bp, NCBI accession number NC_001526) using the Torrent Mapping Alignment Program v5.0.13. SNP calls were made using the Torrent Variant Caller v.5.0.3, and variants were annotated with HPV gene/region using snpEff v.3.6c^87^. Pipeline settings and parameters can be found at https://github.com/cgrlab/cgrHPV16.

### HPV16 variant lineage classification

HPV16 variant lineage assignment was based on the maximum likelihood (ML) tree topology constructed using RAxML MPI v7.2.8.27^88^ that included 16 HPV16 European and non-European variant lineage reference sequences. We excluded samples with overall poor coverage, per individual nucleotide site per sample with low reads (<5), as previously described^24^.

### Statistical analyses

To identify the mutational processes generating the HPV variants, we carried out a de novo mutational signature analysis. There are a total of 12 possible single-nucleotide variants (SNV). The SNVs on the complementary DNA strands are considered the same, and we use pyrimidines (C and T) to annotate the SNV. Therefore, we have the following six basic types of SNVs: C > A, C > G, C > T, G > A, G > C, and G > T. We further considered the adjacent nucleotides in both 5′ and 3′ directions around the SNV as a three base-pair motif and obtained 96 (4 × 6 × 4) mutation types. We calculated the frequency of variants belonging to 96 mutation types for 3579 HPV16 genomes in a 96 × 3579 mutational catalog matrix. The mutational catalog matrix is regarded as a combination of mutational signatures induced by multiple mutational processes. To extract de novo mutational signatures, we applied the non-negative matrix factorization^89^ and compared the similarity of resulting mutational signatures with the COSMIC mutational signatures v3 measured by the cosine similarity.

We calculated the frequency of 96 mutation types, based on a three base-pair motif, across the HPV16 genome. Among these mutation types, APOBEC3-induced mutations are identified as C > T or C > G mutations specifically at the TCW motif (W is A or T), for which only T allowed in the 5′ end and G and C are excluded in the 3′ end^40^. This definition of APOBEC3-induced mutations has been well established and is more stringent than the motif defined (C > T mutations at motif YCN with Y a C or T and N being any nucleotide) by Vartanian et al.^52^.

Both rare (minor allele frequency (MAF) < 0.01) and common (MAF > = 0.01) APOBEC3-induced mutations were examined, and variants occurring in <10% of the sequence reads and the lineage-diagnostic sites^68^ were excluded. For each APOBEC3-induced mutation, we estimated the variant allele frequency (VAF) for HPV16 sequence reads per woman. Note, the difference between MAF and VAF: MAF quantifies the frequency of an APOBEC3-induced mutation across all samples in a given study or among women in a population (regardless of case and control status), while VAF measures the frequency of sequence reads containing the APOBEC3-induced mutation among all reads covering a specific APOBEC3 motif within a sample or per woman. Hence, the VAF reflects the percentage of HPV viruses, or sequence reads, with the specific APOBEC3-induced mutation in a sample. The VAF is expected to be ~1.0, if the APOBEC3-induced mutation was present in the virus at acquisition and being replicated subsequently; in contrast, the VAF would be much lower if the APOBEC3-induced mutation occurs de novo during the infection period, and the virus with this somatic mutation has not become dominant in the sample.

We defined “high VAF” as a variant occurring in >60% of the sequence reads, which we have previously published performs well for calling the predominant HPV variant for a haploid HPV genome^24^. To evaluate lower level within-host somatic changes, we defined “low VAF” as variants occurring in 10% to < = 60% of sequence reads. To minimize false positive mutation calls, we used a VAF lower cut point of 10% and required at least ten sequence reads for each variant call.

To count the number of APOBEC3 targetable sites, we first counted the TCW motifs across the HPV16 reference A1 genome, and for each main HPV16 sublineage. Since there are three possible changes at each nucleotide position (C > A, C > T and C > G), APOBEC3 targetable sites for the C > T and C > G changes only at TCW motif were counted as two-thirds of the number of TCW motifs.

To examine the relative contribution of APOBEC3A- and APOBEC3B-induced mutations across the HPV genome, we compared the proportion of APOBEC3A mutations and APOBEC3B mutations separately by their specific motifs. APOBEC3A and APOBEC3B specific motifs have been reported as distinguishable in the yeast genome as YTCA (for APOBEC3A) and RTCA (for APOBEC3B) (where Y is a pyrimidine base and R is a purine base)^67^.

We examined the association between the presence or absence of mutations with case and control status for all samples.

Logistic regression was used to obtain the odds ratio (OR) and 95% confidence intervals (CI) for precancer/cancer risk for the specified exposure groups using the controls (i.e., women with HPV16 and <CIN2) as the referent group. A chi-squared test was used to compare the distribution of women having an APOBEC3-induced mutation (per individual, coded as “yes” at least one APOBEC3 mutation or “no” APOBEC3 mutations) among HPV16 sublineages.

For a subset of samples with at least one APOBEC3-induced mutation, we further compared the mutation burden of APOBEC3-induced mutations per virus between nonsynonymous and synonymous variants and between cases and controls, which adjusts the sample size of the cases and controls and the potential mutable bases of APOBEC3-induced nonsynonymous and synonymous mutations, respectively. Let *Y*_*ijk*_ represent the number of synonymous (denoted by *j* = 1) or nonsynonymous (*j* = 2) APOBEC3-induced mutations in cases (*i* = 2) and controls (*i* = 1) for each type of APOBEC3-induced mutation (*k* = 1, 2…, 8). The *Y*_*ijk*_’s are modeled by a Poisson distribution with expected count *E*(*Y*_*ijk*_): (1) $E(Y_{11k}) = N_{11k} \times t$ with $N_{11k}$ the number of potential mutable bases and *t* the mutation burden for synonymous mutations in controls; (2) $E(Y_{12k}) = N_{12k} \times t \times w$ with $w = \frac{{\frac{{E(Y_{12})}}{{N_{12}}}}}{{\frac{{E(Y_{11})}}{{N_{11}}}}}$ the mutation burden ratio of nonsynonymous and synonymous mutations in controls, where $E(Y_{12}) = \mathop {\sum}\nolimits_{k = 1}^8 E (Y_{12k})$and $N_{12} = \mathop {\sum}\nolimits_{k = 1}^8 {N_{12k}}$ and similar notations hold for *E*(*Y*_11_) and *N*_11_; (3) *E*(*Y*_21*k*_) = *N*_21*k*_ × *r*_syn_ with $r_{{\mathrm{syn}}} = \frac{{\frac{{E(Y_{21})}}{{N_{21}}}}}{{\frac{{E(Y_{11})}}{{N_{11}}}}}$ the enrichment of synonymous mutations in cases compared to controls, where $E(Y_{21}) = \mathop {\sum}\nolimits_{k = 1}^8 E (Y_{21k})$ and $N_{21} = \mathop {\sum}\nolimits_{k = 1}^8 {N_{21k}}$; and (4) *E*(*Y*_22*k*_) = *N*_22*k*_ × *t* × *w* *r*_nonsyn_ with $r_{{\mathrm{nonsyn}}} = \frac{{\frac{{E(Y_{22})}}{{N_{22}}}}}{{\frac{{E(Y_{12})}}{{N_{12}}}}}$ mutation burden ratio or the enrichment of nonsynonymous mutations in cases compared to controls, where $E(Y_{22}) = \mathop {\sum}\nolimits_{k = 1}^8 E (Y_{22k})$ and $N_{22} = \mathop {\sum}\nolimits_{k = 1}^8 {N_{22k}}$ and similar notations hold for *E*(*Y*_12_) and *N*_12_. *w* is essentially the *d*_N_/*d*_S_ ratio measuring the selection of nonsynonymous mutations compared to synonymous mutations in controls; if *w* = 1, it suggests that the mutations are neutral, while *w* < 1 suggests that nonsynonymous mutations are under negative or purifying selection. A Poisson regression model was fit to obtain the maximum likelihood estimation (MLE) of *t*, *w*, *r*_syn_, and *r*_nonsyn_.

Statistical analyses were performed with R version 3.5.1; all statistical tests were two-sided.

To determine if APOBEC3-induced mutations contributed to the evolution of HPV16 lineages, we conducted the following analyses: (1) ancestral HPV16 sequences and our current day HPV16 genome sequences were used to create a phylogenetic tree that represents HPV16 ancestral states (Supplementary Fig. 4). Ancestral HPV16 sequences included nine HPV16 lineage/sublineages sequences (All, A, A1, A4, B1, C1, D, D2, and D3) that were inferred using the Maximum Likelihood method^90^ under the Tamura-Nei model^91^. The initial tree was inferred using a pre-computed tree file. The rates among sites were treated as being uniform among sites (Uniform rates option). The analysis included 63 nucleotide reference sequences from R.D.B. All positions containing gaps and missing data were eliminated. There were a total of 7697 HPV16 genome positions in the final data set. We also utilized 239 current day HPV16 sequences that represented A1, A4, B1, C1, D2, and D3 sublineages. All current day HPV16 sequences were controls from the PaP cohort. Evolutionary analyses were conducted in MEGA7^92^. (2) For non-A1 lineages, we selected common HPV16 variant positions that are known lineage-defining positions. For the A1 sublineage, which is the reference sublineage (i.e., there are no lineage-defining positions), we evaluated all common variants (MAF > 1%) occuring within the A1 sublineage viruses. (3) We aligned each ancestral sequence to an ancestral sequence from the previous node in the phylogenetic tree. For example, ancestral A1 was compared with the ancestral A sequence, and ancestral D2 was compared with the ancestral D sequence. Then we calculated the percentage of mutations that were potentially induced by APOBEC3 among lineage-defining positions of that particular lineage or sublineage. (4) When comparing current day HPV16 sequences to the ancestral sequences, we looped over all available current day sequences of that particular sublineage and calculated the average percentage of APOBEC3-induced mutation among lineage-defining positions.

### Reporting summary

Further information on research design is available in the Nature Research Reporting Summary linked to this article.

## Supplementary information

Supplementary Information

Reporting Summary

## Data Availability

The HPV sequencing data have been deposited in the Genbank database under the accession codes MG847621-MG850835. All the other data supporting the findings of this study are available within the article and its supplementary information files and from the corresponding author upon reasonable request. A reporting summary for this article is available as a Supplementary Information file.

## Code availability

The software and algorithms used for our analyses are specified in the corresponding methods section. Pipeline settings and parameters used to call HPV16 variants can be found at https://github.com/cgrlab/cgrHPV16.
