# Supplementary material for: Mutations in the HPV16 genome induced by APOBEC3 are associated with viral clearance
Source: Nat Commun. 2020 Feb 14;11:886. doi: 10.1038/s41467-020-14730-1 (PMC7021686; doi:10.1038/s41467-020-14730-1)
Supplement: Supplementary file 1 — Supplementary Information [file 41467_2020_14730_MOESM1_ESM.pdf]

Supplementary Information

Mutations in the HPV16 genome induced by APOBEC3 are associated with viral clearance

Zhu et al.

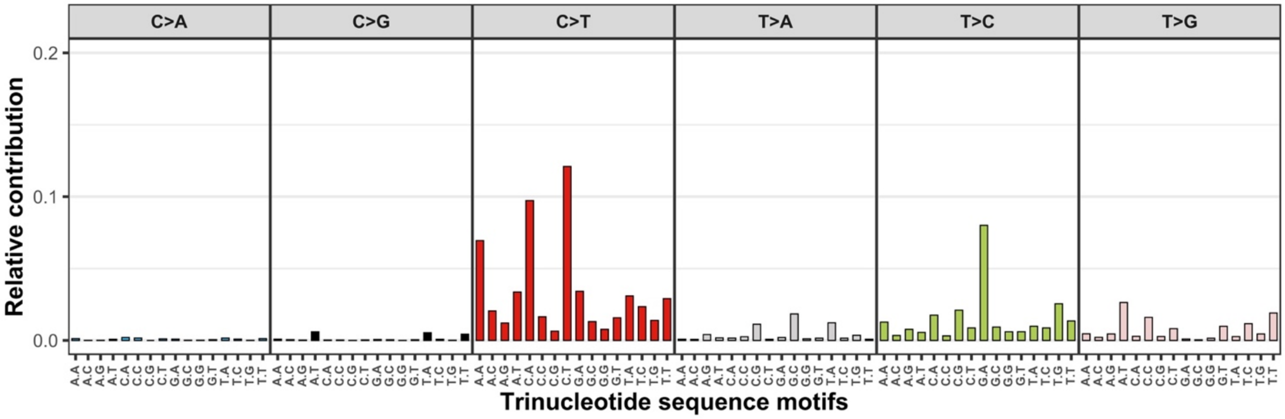

Supplementary Figure 1: Frequency of the 96 trinucleotide mutation types for variants across the HPV16 genome in women from the PaP cohort. The x-axis indicates the 5' and 3' nucleotides for each of the top panel substitutions for the 3 base-pair motifs.

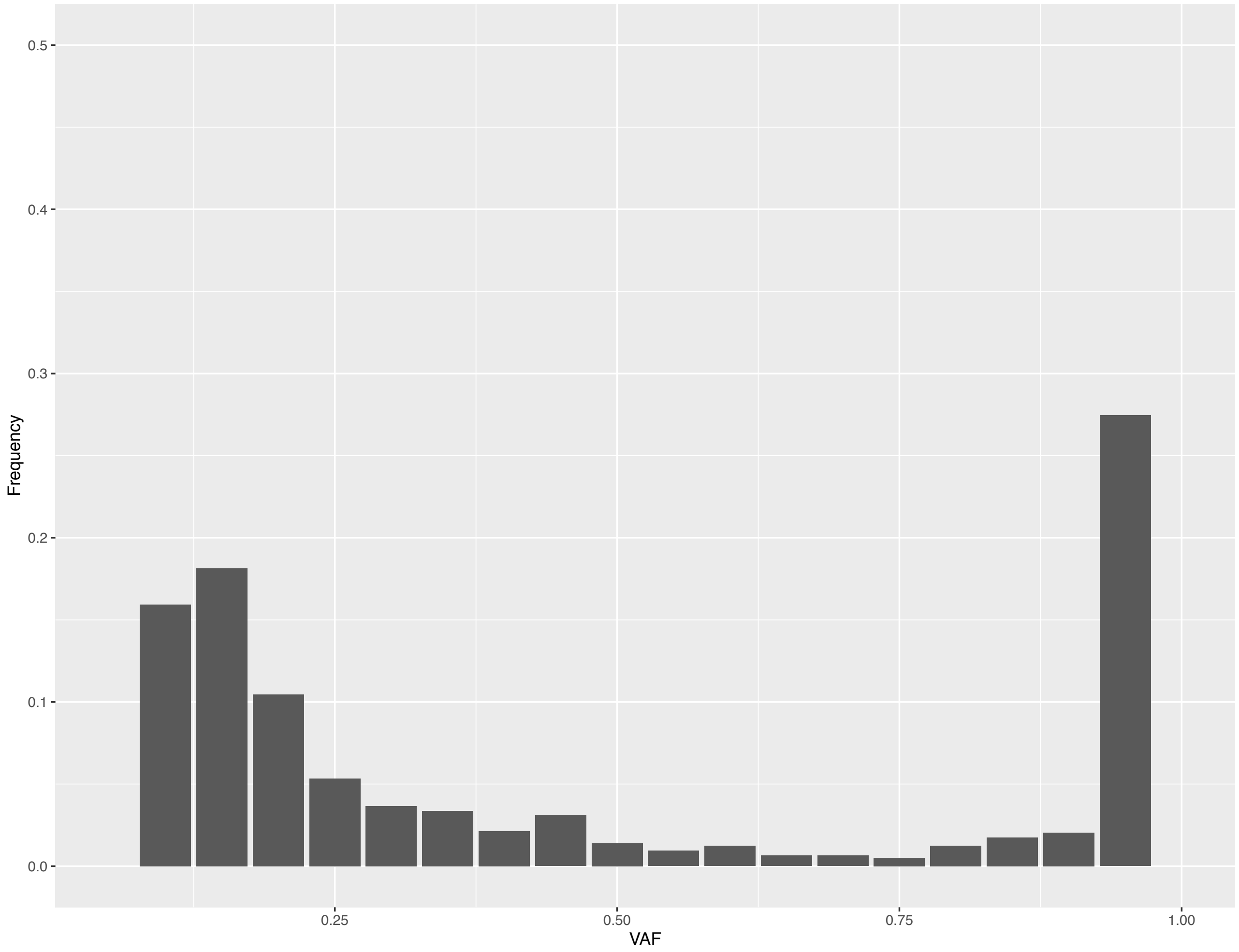

Supplementary Figure 2: Frequency distribution of APOBEC3-induced mutations across the HPV16 genome by variant allele fraction (VAF) for women in the PaP cohort. The APOBEC3-induced mutations are categorized into 18 groups based on their VAFs and each bar shows the proportion of APOBEC3-induced mutations belonging to the VAF group.

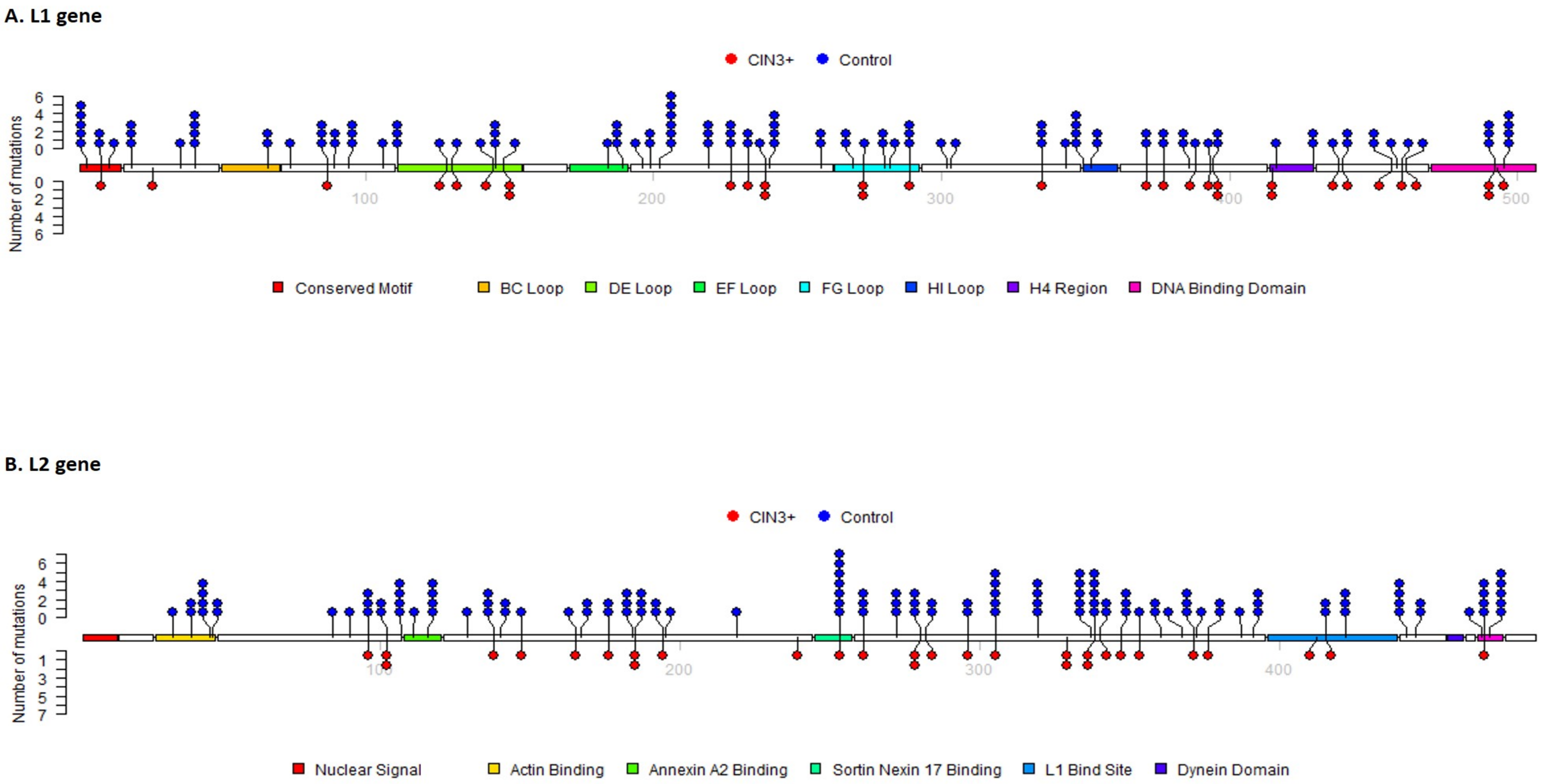

Supplementary Figure 3: Somatic low VAF APOBEC3-induced mutations observed in the (A) L1 and (B) L2 ORF in the CIN3+ cases (red lollipops) and controls (blue lollipops) in women from the PaP cohort. The domains of L1 and L2 are colored, see legend. Cases are cervical intraepithelial neoplasia grade 3 and cancer cases (CIN3+). The number of circles shown with each variant indicate the number of individuals with that variant.

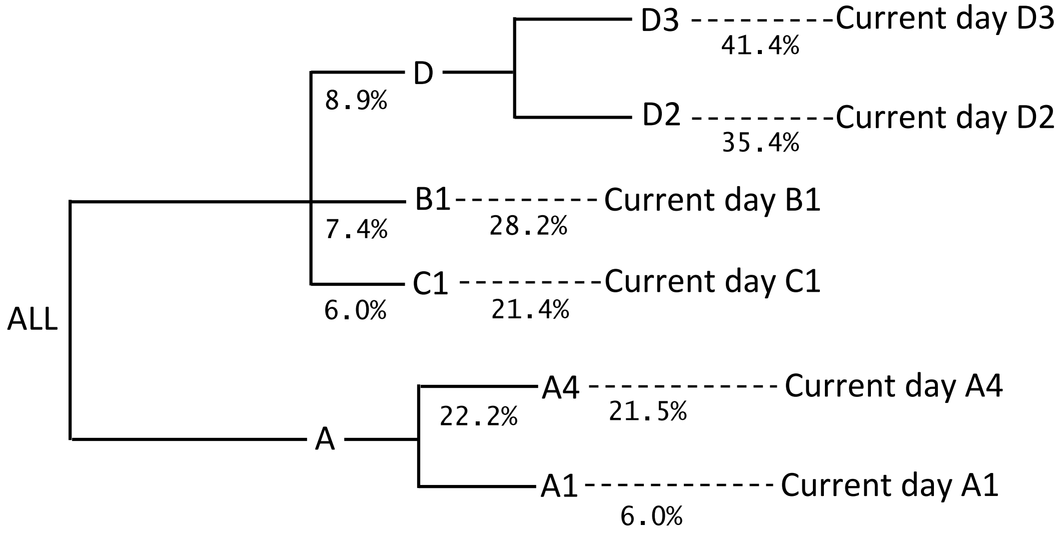

Supplementary Figure 4: APOBEC3-induced mutations contributed to the evolution of viral lineages. HPV16 phylogenetic tree illustrating each main sublineage (A1, A4, C1, B1, D2, D3) and the percentage of the lineage-defining sites that were potentially induced by APOBEC3 compared with the ancestral sequence at each node of the tree.

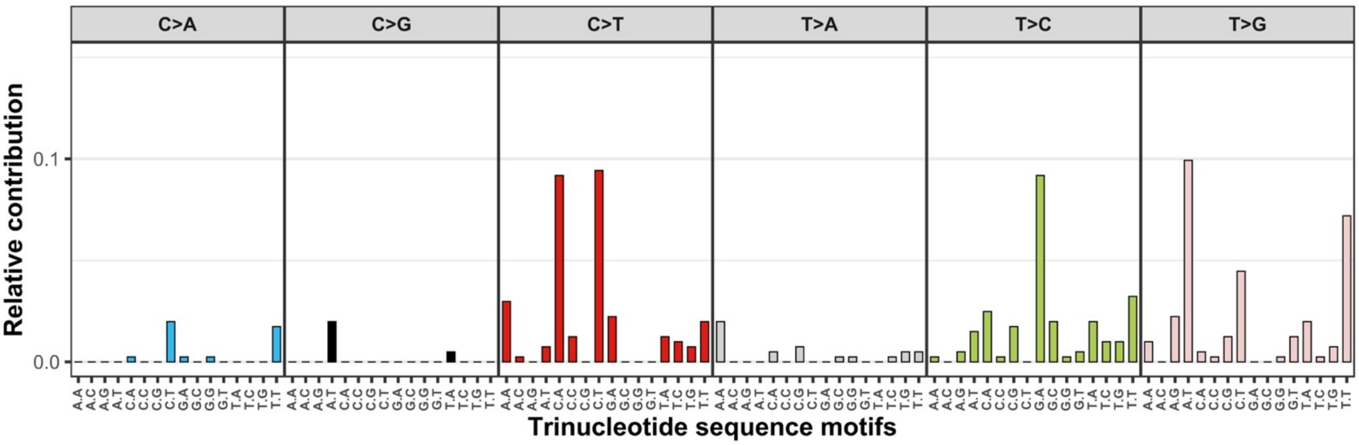

Supplementary Figure 5: Frequency of the 96 trinucleotide mutation types for variants across 45 HPV16 genomes sequenced by Hirose et al., J Virol 2018:92;e00017-00018. HPV16 genome sequence data retrieved from GenBank, accession numbers: LC368952 to LC368996. The common evolutionary-derived HPV16 lineage-defining variants are removed.

Supplementary Table 1. Number of women with and without APOBEC3-induced mutations stratified by variant allele fraction (VAF) in CIN2+ cases and controls of the NCI-Kaiser PaP cohort.

VAF	Status	No APOBEC3 mutation	APOBEC3 mutation	%	OR	95% CI	*P*-value[1]
**Low[2]**	CIN2+	2023	291	12.6%	0.48	(0.40, 0.57)	5.8×10^{-16}
	Control	971	294	23.2%	ref		
**High[3]**	CIN2+	1840	474	20.5%	1.03	(0.87, 1.23)	0.76
	Control	1012	253	20.0%	ref		

CIN2+, cervical intraepithelial neoplasia grade 2 and grade 3, and cancer cases; OR, odds ratio; CI, confidence intervals; ref, referent group;

[1] Fisher's exact test, two-sided, comparing the number of women with at least one APOBEC3-induced mutation and those without APOBEC3-induced mutations in the CIN2+ cases to controls;

[2] Low VAF is defined as VAF >10% and <=60%;

[3] High VAF is defined as VAF >60%.

Supplementary Table 2. Burden of APOBEC3-induced mutations in CIN3+ cases vs. controls and nonsynonymous vs. synonymous mutations by variant allele fraction (VAF) in the NCI-Kaiser PaP cohort.

VAF	Parameter	Interpretation	Viral gene	Mutation burden[1]	95% CI	FDR adjusted *P*-value[2]
**Low[3]**	r_{nonsyn}	Enrichment of nonsynonymous mutations in cases vs. controls by gene region	*E6*	1.13	(0.70, 1.81)	0.70
			E7	0.55	(0.27, 1.11)	0.16
			E1	0.72	(0.54, 0.96)	0.06
			E2	1.07	(0.79, 1.45)	0.71
			E4	1.01	(0.49, 2.09)	0.99
			E5	0.35	(0.08, 1.58)	0.22
			L2	0.46	(0.31, 0.68)	6.7×10^{-4}
			L1	0.54	(0.36, 0.80)	0.01
**High[4]**	r_{nonsyn}	Enrichment of nonsynonymous mutations in cases vs. controls by gene region	*E6*	0.64	(0.37, 1.09)	0.17
			E7	0.22	(0.05, 1.05)	0.12
			E1	0.94	(0.58, 1.54)	0.82
			E2	1.14	(0.76, 1.71)	0.63
			E4	7.99	(1.85, 34.43)	0.01
			E5	1.15	(0.51, 2.63)	0.82
			L2	0.75	(0.55, 1.03)	0.14
			L1	0.65	(0.33, 1.30)	0.30

CIN3+, cervical intraepithelial neoplasia grade 3 and cancer cases; CI, confidence intervals;

[1] Mutation burden ratio of APOBEC3-induced mutations was calculated using a Poisson regression model to compare the mutation burden or enrichment of APOBEC3-induced mutations per virus between cases and controls for nonsynonymous mutations (r) by viral gene region;

[2] False discovery rate (FDR) adjusted P-values; P-values are generated by a Wald test of the Poisson regression model;

[3] Low VAF is defined as VAF >10% and <=60%;

[4] High VAF is defined as VAF >60%.

Supplementary Table 3. Number of women with and without APOBEC3-induced mutations stratified by variant allele fraction (VAF) in SUCCEED CIN3+ cases and IARC cancer cases.

VAF	Study	Status	No APOBEC3 mutation	APOBEC3 mutation	%	OR[4]	95% CI	*P*-value
Low[2]	SUCCEED	CIN3+	389	55	12.4%	0.47	(0.34, 0.64)	$5.1x10^{-7}$
	IARC	Cancers	1142	163	12.5%	0.47	(0.38, 0.58)	$1.1x10^{-12}$
High[3]	SUCCEED	CIN3+	333	111	25.0%	1.33	(1.02, 1.73)	0.03
	IARC	Cancers	992	313	24.0%	1.26	(1.04, 1.53)	0.02

CIN3+, cervical intraepithelial neoplasia grade 3 and cancer cases; OR, odds ratio; CI, confidence intervals;
[1] Fisher's exact test, two-sided, comparing the number of women with at least one APOBEC3-induced mutation and those without APOBEC3-induced mutations in the cases to controls;
[2] Low VAF is defined as VAF >10% and <=60%;
[3] High VAF is defined as VAF >60%;
[4] Odd ratios, 95% CI and *P*-values, estimated using logistic regression comparing SUCCEED or IARC cases to the PaP controls (referent group).

Supplementary Table 4. Number of women with and without the specified signature-induced mutation at a low variant allele fraction in CIN3+ cases and controls in the NCI-Kaiser PaP cohort.

Mutational signature	Status	No signature mutation	Signature mutation	%	OR	95% CI	*P*-value[4]
**Signature B[1]**	CIN3+	819	463	36.1%	0.74	(0.63, 0.87)	0.0003
	Control	718	547	43.2%	ref		
**Signature C[2]**	CIN3+	1030	252	18.5%	0.50	(0.41, 0.60)	$3.1x10^{-14}$
	Control	849	416	32.9%	ref		
**Signature D[3]**	Case	257	1025	20.0%	0.60	(0.50, 0.72)	$3.4x10^{-8}$
	Control	373	892	29.5%	ref		

CIN3+, cervical intraepithelial neoplasia grade 3 and cancer cases; OR, odds ratio; CI, confidence intervals;
[1] Signature B is defined as C>T mutations outside the TCW motif (excluding C>T mutations in signature A);
[2] Signature C is defined as all T>C mutations;
[3] Signature D is defined as all other mutations not in signatures A, B or C;
[4] Fisher's exact test, two-sided.

Supplementary Table 5. Estimated number of APOBEC3 targetable sites for each HPV16 sublineage genome.

HPV16 Sublineage	TCW motifs	Targetable sites†
A1	394	263
A2	392	261
A4	390	260
B1	388	259
C1	384	256
D2	370	247
D3	369	246

† the number of APOBEC3 targetable sites was estimated as two thirds of the total number of TCW motifs present in each genome since there are three possible changes at each nucleotide position (C>A, C>T and C>G) and APOBEC3 is associated with two changes, C>T and C>G.

Supplementary Table 6. Number of total mutations, stratified by nonsynonymous (nonsyn) and synonymous (syn), at a low variant allele fraction in CIN3+ cases vs. controls in the NCI-Kaiser PaP cohort.

Status	No. of women	All† mutations	All, nonsyn	%	All, syn	APOBEC3‡ mutations	APOBEC3, nonsyn	%	APOBEC3, syn
**CIN3+**	1282	2515	1772	70.5%	743	238	227	95.4%	11
**Control**	1265	5985	4284	71.6%	1701	644	613	95.2%	31
**Total**	2547	8500	6056	71.2%	2444	882	840	95.2%	42

CIN3+, cervical intraepithelial neoplasia grade 3 and cancer cases;
† includes all substitutions observed across the HPV16 genome;
‡ includes only the APOBEC3-induced mutations.

Supplementary Table 7. Number of women with the specified number of APOBEC3-induced nonsynonymous (nonsyn) and synonymous (syn) mutations at a low variant allele fraction in CIN3+ cases vs. controls in the NCI-Kaiser PaP cohort.

**Status**	**Number of APOBEC3-induced mutations**				
	**0**	**1**	**2**	**3**	**4+**
**CIN3+, N=1,282**					
**Nonsyn**	1140	100	20	10	12
**Syn**	1272	9	1	0	0
**Control, N=1,265**					
**Nonsyn**	985	133	64	40	43
**Syn**	1238	23	4	0	0

CIN3+, cervical intraepithelial neoplasia grade 3 and cancer cases.

Supplementary Table 8. Burden of APOBEC3-induced mutations on the positive vs. negative strand.

VAF	Strand	Mutation burden	*P*-value[4]
**Low[1]**	Positive	6.3/kb	0.47
	Negative	6.9/kb	
**High[2]**	Positive	5.0/kb	0.18
	Negative	4.0/kb	
**All[3]**	Positive	5.8/kb	0.61
	Negative	6.2/kb	

[1] Low variant allele fraction (VAF)is defined as VAF>10% and <=60%;
[2] High VAF is defined as VAF >60%;
[3] All VAF is defined as VAF>10%;
[4] Proportion test.

Supplementary Table 9. Burden of APOBEC3-induced mutations for APOBEC3A vs. APOBEC3B possible sites.

VAF	APOBEC3	Mutation burden	*P*-value[4]
**Low[1]**	APOBEC3A	0.33/kb	0.98
	APOBEC3B	0.32/kb	
**High[2]**	APOBEC3A	0.22/kb	0.10
	APOBEC3B	0.15/kb	
**All[3]**	APOBEC3A	0.35/kb	0.86
	APOBEC3B	0.34/kb	

[1] Low variant allele fraction (VAF) is defined as VAF>10% and <=60%;
[2] High VAF is defined as VAF >60%;
[3] All VAF is defined as VAF>10%;
[4] Proportion test.
