# Supplementary material for: Mutations in the HPV16 genome induced by APOBEC3 are associated with viral clearance
Source: Nat Commun. 2020 Feb 14;11:886. doi: 10.1038/s41467-020-14730-1 (PMC7021686; doi:10.1038/s41467-020-14730-1)
Supplement: Supplementary file 2 — Reporting Summary [file 41467_2020_14730_MOESM2_ESM.pdf]

nature research

Corresponding author(s): Lisa Mirabello

Last updated by author(s): Jun 3, 2019

Reporting Summary

Nature Research wishes to improve the reproducibility of the work that we publish. This form provides structure for consistency and transparency in reporting. For further information on Nature Research policies, see Authors & Referees and the Editorial Policy Checklist .

Statistics

For all statistical analyses, confirm that the following items are present in the figure legend, table legend, main text, or Methods section.

n/a	Confirmed	
☐	☒	The exact sample size (*n*) for each experimental group/condition, given as a discrete number and unit of measurement
☐	☒	A statement on whether measurements were taken from distinct samples or whether the same sample was measured repeatedly
☐	☒	The statistical test(s) used AND whether they are one- or two-sided<br>*Only common tests should be described solely by name; describe more complex techniques in the Methods section.*
☐	☒	A description of all covariates tested
☐	☒	A description of any assumptions or corrections, such as tests of normality and adjustment for multiple comparisons
☐	☒	A full description of the statistical parameters including central tendency (e.g. means) or other basic estimates (e.g. regression coefficient) AND variation (e.g. standard deviation) or associated estimates of uncertainty (e.g. confidence intervals)
☐	☒	For null hypothesis testing, the test statistic (e.g. *F*, *t*, *r*) with confidence intervals, effect sizes, degrees of freedom and *P* value noted<br>*Give P values as exact values whenever suitable.*
☒	☐	For Bayesian analysis, information on the choice of priors and Markov chain Monte Carlo settings
☒	☐	For hierarchical and complex designs, identification of the appropriate level for tests and full reporting of outcomes
☐	☒	Estimates of effect sizes (e.g. Cohen's *d*, Pearson's *r*), indicating how they were calculated

Our web collection on statistics for biologists contains articles on many of the points above.

Software and code

Policy information about availability of computer code

Data collection	No software is used in data collection
Data analysis	Statistical analyses were performed with R version 3.5.1; all statistical tests were two-sided.

For manuscripts utilizing custom algorithms or software that are central to the research but not yet described in published literature, software must be made available to editors/reviewers. We strongly encourage code deposition in a community repository (e.g. GitHub). See the Nature Research guidelines for submitting code & software for further information.

Data

Policy information about availability of data

All manuscripts must include a data availability statement. This statement should provide the following information, where applicable:

- Accession codes, unique identifiers, or web links for publicly available datasets
- A list of figures that have associated raw data
- A description of any restrictions on data availability

HPV genome sequence data is publicly available in Genbank, accession numbers MG847621-MG850835

Field-specific reporting

Please select the one below that is the best fit for your research. If you are not sure, read the appropriate sections before making your selection.

☒ Life sciences ☐ Behavioural & social sciences ☐ Ecological, evolutionary & environmental sciences

For a reference copy of the document with all sections, see nature.com/documents/nr-reporting-summary-flat.pdf

Life sciences study design

All studies must disclose on these points even when the disclosure is negative.

Sample size	Total sample size is 5,328 HPV16-positive women.
Data exclusions	We included all individuals that were HPV16-positive with cervical precancer or cancer in our studies, and matched an approximate number of HPV16-positive controls with benign infections in our NCI PaP cohort. After sequencing, we excluded samples with overall poor coverage, per individual nucleotide site per sample with low reads (<5).
Replication	Two case studies were used as replication of case findings: 444 precancers/cancers from our NCI SUCCEED study, and 1,305 cancers collected worldwide with IARC.
Randomization	All cases were selected and controls randomly selected to approximately equal numbers of precancer.
Blinding	The laboratory was blinded to case-control status for HPV genome sequencing.

Reporting for specific materials, systems and methods

We require information from authors about some types of materials, experimental systems and methods used in many studies. Here, indicate whether each material, system or method listed is relevant to your study. If you are not sure if a list item applies to your research, read the appropriate section before selecting a response.

Materials & experimental systems

n/a	Involved in the study
☒	☐ Antibodies
☒	☐ Eukaryotic cell lines
☒	☐ Palaeontology
☒	☐ Animals and other organisms
☐	☒ Human research participants
☒	☐ Clinical data

Methods

n/a	Involved in the study
☒	☐ ChIP-seq
☒	☐ Flow cytometry
☒	☐ MRI-based neuroimaging

Human research participants

Policy information about studies involving human research participants

Population characteristics	See above.
Recruitment	For our NCI PaP cohort, cases and controls were chosen from the Kaiser Permanente Northern California (KPNC)-NCI HPV Persistence and Progression cohort, which is a repository of residual cervical specimens from women who underwent cervical cancer screening from January 2007 to January 2011 at KPNC. Women could opt-out of having their residual cervical specimens retained; only 8% of women with collected specimens opted out from having their specimen banked and tested. De-identified demographic and clinical information as well as all HPV and cytology test results and cervical histopathology were obtained on the cohort from electronic health records.<br>For the NCI SUCCEED study, women were enrolled into SUCCEED between November 2003 and October 2009. We recruited women that were referred to colposcopy or treatment at the University of Oklahoma Dysplasia Clinic based at the University of Oklahoma Health Sciences Centre (OUHSC), with a recent abnormal Pap smear diagnosis or a biopsy diagnosis of CIN/cancer.<br>For IARC cancers, HPV16-positive cervical cell or tissue (frozen biopsy or formalin-fixed paraffin-embedded [FFPE]) specimens from cervical cancer cases were part of the IARC-coordinated cervical cancer case series, cervical cancer case–control studies and population-based HPV prevalence surveys from 39 countries worldwide.
Ethics oversight	The PaP study protocol was reviewed and approved yearly by Kaiser Permanente and the National Cancer Institute Institutional Review Boards.<br>For SUCCEED, written informed consent was obtained from all women enrolled in the study and Institutional Review Board approval was provided by OUHSC and the US National Cancer Institute.<br>For IARC samples, both local and IARC ethical committees approved all studies.

Note that full information on the approval of the study protocol must also be provided in the manuscript.
